# Supplementary material for: Nuclear Expression of TDP-43 Is Linked with Morphology and Ubiquitylation of Cytoplasmic Aggregates in Amyotrophic Lateral Sclerosis
Source: Int J Mol Sci. 2023 Jul 29;24(15):12176. doi: 10.3390/ijms241512176 (PMC10418808; doi:10.3390/ijms241512176)
Supplement: Supplementary file 1 [file ijms-24-12176-s001.zip › ijms-2510835-supplementary.pdf]

Table S1. tissue availability

																					TDP-43 pathology in LMN system			
	disease		XII CN																		n/c-ir in	c-ir in	n/c-ir in	c-ir in
	duration	PMC	nucleus	cervical cord					thoracic cord						lumbar cord					sacral cord	neuron	neuron	glia	glia
	month			C4	C5	C6	C7	C8	T1	T2	T3	T4	T5	T6	L1	L2	L3	L4	L5	S2				
case 1	6	yes	yes	yes	yes	yes	yes	yes	yes	yes	yes	yes	yes	yes	yes	yes	yes	yes	yes	yes	34	31	10	1
case 2	8	yes	yes	yes	yes	yes	yes	yes	yes	yes	yes	yes	yes	yes	yes	yes	yes	yes	yes	yes	9	67	112	1
case 3	9	yes	yes	yes	yes	yes	yes	yes	yes	yes	yes	yes	yes	yes	yes	yes	yes	yes	yes	yes	7	58	252	2
case 4	12	yes	yes	NA	NA	NA	yes	yes	yes	yes	yes	yes	yes	yes	yes	NA	yes	yes	yes	yes	4	44	15	1
case 5	12	yes	yes	yes	yes	yes	yes	yes	yes	yes	yes	yes	yes	yes	yes	yes	yes	yes	yes	yes	0	4	98	2
case 6	12	yes	yes	yes	NA	yes	yes	yes	yes	yes	yes	yes	yes	yes	yes	yes	yes	NA	NA	yes	6	44	65	0
case 7	13	yes	yes	yes	yes	NA	yes	yes	yes	yes	yes	yes	yes	yes	yes	yes	yes	yes	yes	yes	6	38	105	5
case 8	15	yes	yes	yes	yes	yes	yes	yes	NA	yes	yes	yes	yes	yes	yes	yes	yes	yes	yes	yes	4	50	26	0
case 9	15	yes	yes	NA	yes	yes	yes	yes	yes	yes	yes	yes	yes	yes	yes	yes	yes	yes	yes	yes	3	30	36	4
case 10	18	yes	yes	yes	yes	yes	yes	yes	yes	yes	yes	yes	yes	yes	yes	NA	yes	yes	yes	yes	1	19	34	0
case 11	18	yes	yes	yes	yes	NA	yes	yes	yes	yes	yes	yes	yes	yes	yes	yes	yes	yes	yes	yes	25	80	25	2
case 12	20	yes	yes	yes	NA	NA	NA	NA	yes	yes	yes	yes	yes	yes	yes	yes	yes	yes	yes	yes	7	24	176	6
case 13	22	yes	yes	yes	yes	yes	yes	yes	yes	yes	yes	yes	yes	yes	yes	yes	yes	yes	yes	yes	11	26	51	0
case 14	28	yes	yes	yes	yes	yes	yes	yes	yes	yes	yes	yes	yes	yes	yes	NA	NA	yes	yes	yes	3	33	126	1
case 15	31	yes	yes	yes	yes	NA	yes	yes	yes	yes	yes	yes	yes	yes	yes	NA	yes	yes	yes	yes	4	43	106	0
case 16	40	yes	yes	yes	yes	yes	yes	yes	yes	yes	yes	yes	yes	yes	yes	NA	yes	yes	yes	yes	6	15	21	1
case 17	49	yes	yes	yes	yes	yes	yes	yes	yes	yes	yes	yes	yes	yes	yes	yes	yes	yes	yes	yes	0	15	16	0
case 18	55	yes	yes	NA	yes	yes	yes	yes	NA	yes	yes	yes	yes	yes	yes	yes	NA	yes	yes	yes	0	8	9	0
case 19	60	yes	yes	yes	NA	yes	yes	yes	yes	yes	yes	yes	yes	yes	yes	yes	yes	yes	yes	yes	0	9	175	2
case 20	64	yes	yes	yes	yes	yes	yes	yes	yes	yes	yes	yes	yes	yes	yes	yes	yes	yes	yes	yes	0	10	77	0
case 21	84	yes	yes	yes	NA	yes	yes	yes	yes	yes	yes	yes	yes	yes	yes	yes	yes	yes	yes	yes	0	7	65	1
case 22	132	yes	yes	yes	yes	yes	yes	yes	yes	yes	yes	yes	yes	yes	yes	NA	yes	yes	yes	yes	0	5	30	0
total																					130	660	1630	29

LMN lower motor neuron, NA not available, PMC primary motor cortex, XII CN XIIth cranial nerve (hypoglossal nerve)

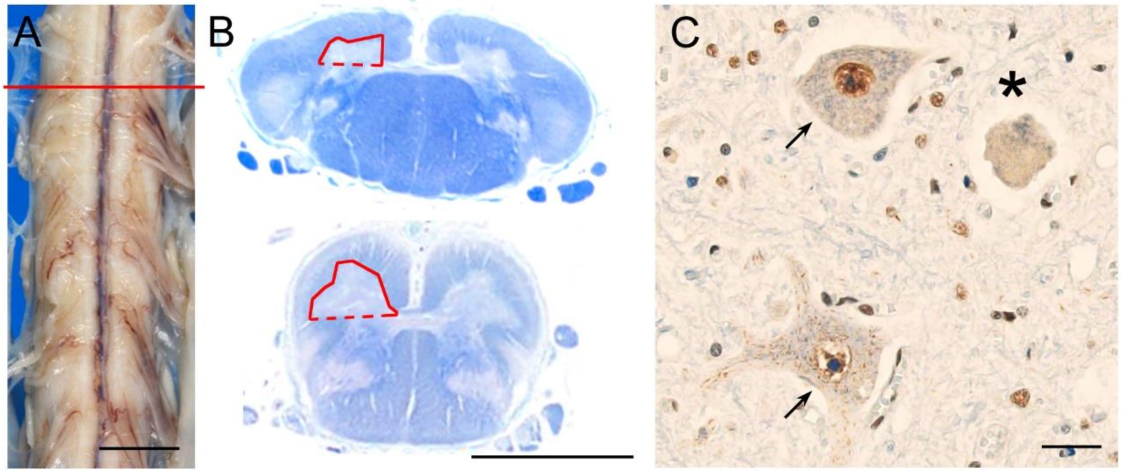

Figure S1. Tissue preparation and neuron counting protocol.

(A) We transversely cut the spinal cord at the most rostral point where the anterior roots exit the cord parenchyma (a red line). (B) In this study, the front border of the anterior horn was defined as the boundary between the anterior column and gray matter (solid red curves). The dorsal border of the anterior horn was defined by a line connecting the most dorsal point of the medial anterior column and the most lateral point of the anterior gray matter (broken red lines). (C) We counted neurons in which nuclei were present on the slide (arrows), whereas we dropped those without visible nuclei (asterisk). Scale bars (A and B) 5 mm and (C) 10 μm.

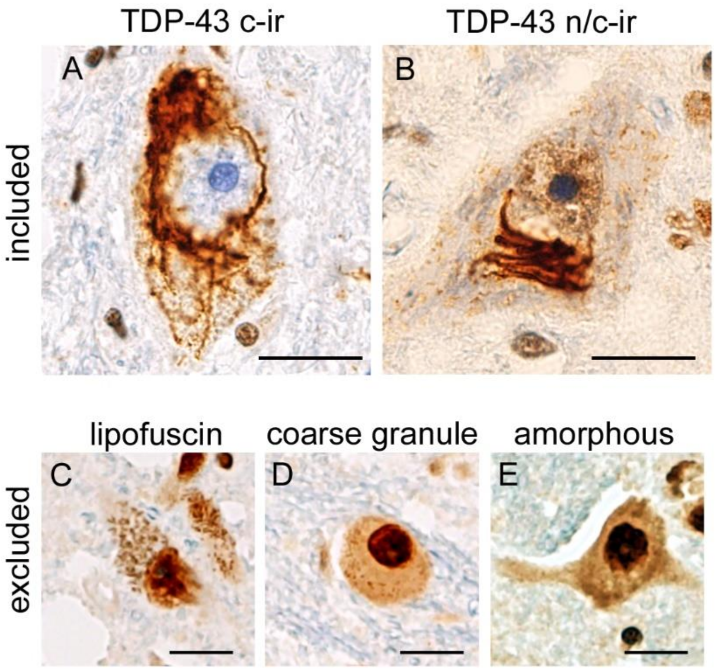

Figure S2. Inclusion and exclusion criteria for TDP-43 aggregates.

Cytoplasmic TDP-43 aggregates with nuclear clearance were defined as TDP-43 c-ir (cytoplasmic immunoreactivity) (A), whereas those combined with nuclear expression were defined as TDP-43 n/c-ir (nuclear and cytoplasmic immunoreactivity) (B). Lipofuscin (C), coarse granular (D), and amorphous diffuse staining (E) of the cytoplasm was observed in both ALS-TDP cases and control subjects, suggesting nonspecific reactions. Anti-TDP-43 immunohistochemistry. Scale bars = 10 μm.

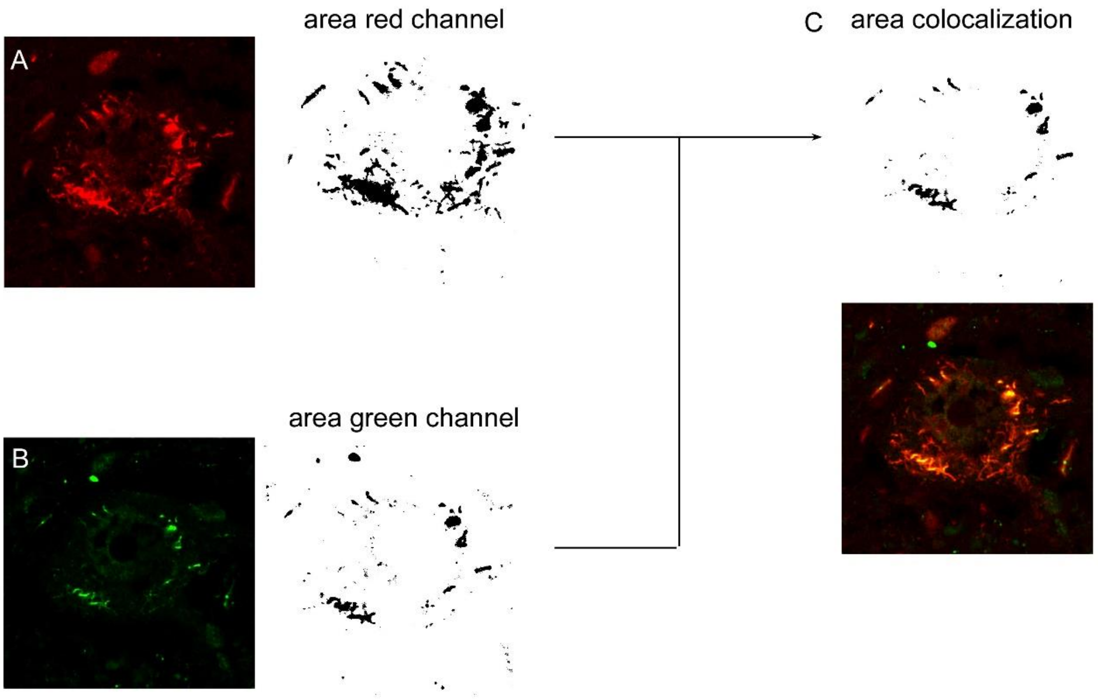

Figure S3. Colocalization analysis. We took photomicrographs under ×1000 magnification. For each neuron, fluorescent signals from red (A) and green (B) channels were automatically detected using the Otsu threshold of ImageJ. Pixels shared between red and green signal areas (C) were automatically detected with the `AND` operation of the software, followed by measurement of the colocalized area.

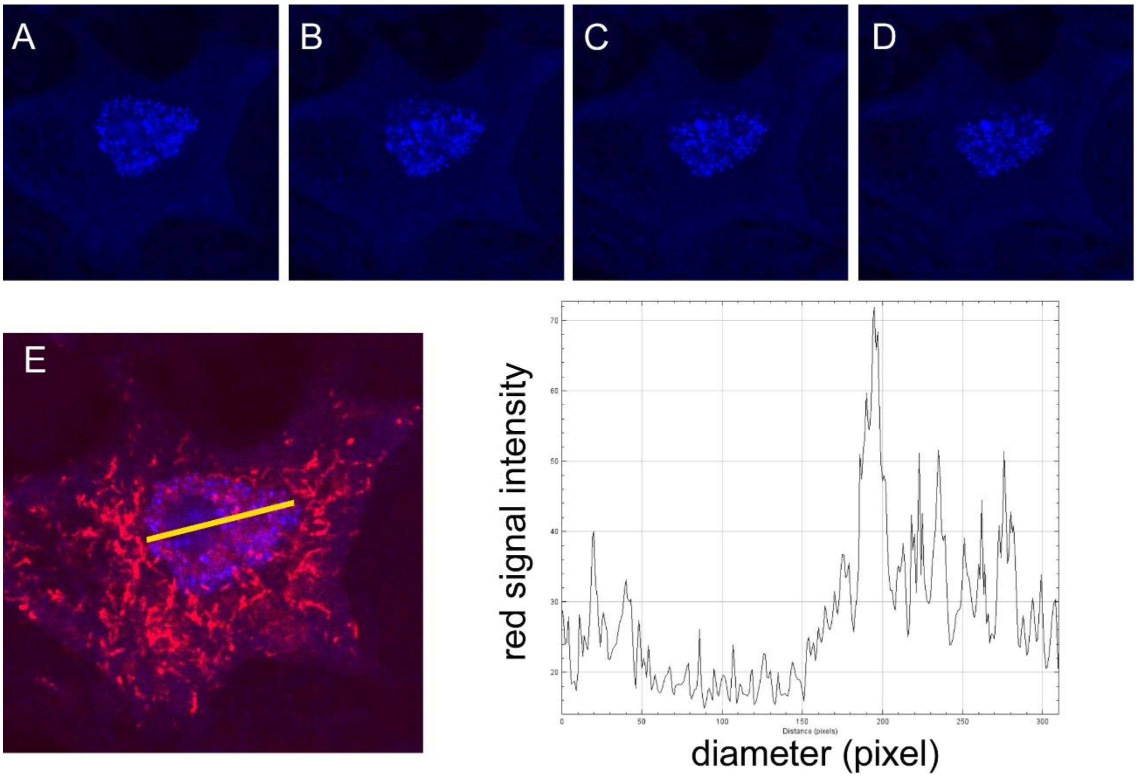

Figure S4. Measurement of fluorescent signal intensity.

(A-D) A stacked image was generated for each neuron. The slice where the nucleus most largely appeared was assessed. (E) We identified the longest diameter running across the nucleus on the basis of DAPI staining, and signal intensities of the red channel (TDP-43) were measured along the nuclear diameter. For each neuron, we computed an averaged signal intensity as [(total signal intensity value)/pixel counts along the diameter], which was defined as the nuclear signal intensity of the neuron.
